# The role of imaging and sentinel lymph node biopsy in patients with T3b-T4b melanoma with clinically negative disease

**DOI:** 10.3389/fonc.2023.1143354

**Published:** 2023-05-08

**Authors:** Marianna V. Papageorge, Renee M. Maina, Amber Loren O. King, Victor Lee, Raymond Baumann, Darko Pucar, Stephan Ariyan, Sajid A. Khan, Sarah A. Weiss, James Clune, Kelly Olino

**Affiliations:** ^1^ Department of Surgery, Yale University School of Medicine, New Haven, CT, United States; ^2^ Department of Surgery, The University of Tennessee Health Science Center, Memphis, TN, United States; ^3^ Yale University School of Medicine, New Haven, CT, United States; ^4^ Department of Pharmacology, Yale University School of Medicine, New Haven, CT, United States; ^5^ Department of Radiology, Yale University School of Medicine, New Haven, CT, United States; ^6^ Rutgers Cancer Institute of New Jersey, Medical Oncology, New Brunswick, NJ, United States

**Keywords:** high-risk melanoma, cross-sectional imaging, surgical management, sentinel lymph node (SLN) biopsy, recurrence free survival

## Abstract

**Background:**

Previous studies demonstrate minimal utility of pre-operative imaging for low-risk melanoma; however, imaging may be more critical for patients with high-risk disease. Our study evaluates the impact of peri-operative cross-sectional imaging in patients with T3b-T4b melanoma.

**Methods:**

Patients with T3b-T4b melanoma who underwent wide local excision were identified from a single institution (1/1/2005 – 12/31/2020). Cross-sectional imaging was defined as body CT, PET and/or MRI in the perioperative period, with the following findings: in-transit or nodal disease, metastatic disease, incidental cancer, or other. Propensity scores were created for the odds of undergoing pre-operative imaging. Recurrence free survival was analyzed using the Kaplan-Meier method and log-rank test.

**Results:**

A total of 209 patients were identified with a median age of 65 (IQR 54-76), of which the majority were male (65.1%), with nodular melanoma (39.7%) and T4b disease (47.9%). Overall, 55.0% underwent pre-operative imaging. There were no differences in imaging findings between the pre- and post-operative cohorts. After propensity-score matching, there was no difference in recurrence free survival. Sentinel node biopsy was performed in 77.5% patients, with 47.5% resulting in a positive result.

**Conclusion:**

Pre-operative cross-sectional imaging does not impact the management of patients with high-risk melanoma. Careful consideration of imaging use is critical in the management of these patients and highlights the importance of sentinel node biopsy for stratification and decision making.

## Introduction

The incidence of primary cutaneous melanoma continues to increase in the United States, with prognosis largely dependent on tumor characteristics such as thickness and the presence or absence of nodal involvement (1, 2). Although the 5-year melanoma specific survival rate for stage I-IIA (T1a/b, T2a/b, T3a) cutaneous melanoma ranges from 94-99%, these numbers drop to 87% for stage IIB (T3b) (>2-4 mm with ulceration) and 82% for T4b (>4 mm with ulceration) melanoma, with recurrence rates as high as 24% (3–5). In fact, patients with thick and/or ulcerated tumors (T3b-T4b) at the time of initial biopsy are at high risk of developing, or already having, locoregional or metastatic disease spread (4). Although sentinel lymph node (SLN) biopsy is a procedure offered to these patients that gives important prognostic information, there are studies indicating that there may be differences in draining dermal lymphatics, particularly in older patients (6, 7). These patients may be less likely to develop nodal disease and but still carry a risk of developing distant disease and this has been reflected in nomograms developed in the United States and Australia (8, 9). Additionally, these older patients harbor additional medical comorbidities which may expose them to a greater risk under general anesthesia that may preclude SLN biopsy, making additional pre-operative staging of increased value in the medical decision-making process (10, 11).

Among stage I-II cutaneous melanoma, the use of positron emission tomography (PET) scans has not resulted in findings ultimately impacting care and is not recommended (12). However, there has been continued interest in the use of pre-operative imaging among patients with stage III-IV melanoma to guide multi-disciplinary clinical decision making. It has been previously demonstrated that cross-sectional pre-operative imaging may provide useful information on disease staging (13). These results become particularly relevant with the recent approval of adjuvant therapy for stage IIB and IIC melanoma, which will likely motivate more widespread use of pre-operative or early post-operative imaging in the treatment planning of these patients, and in certain circumstances may influence utilization of SLN biopsy (14). Additionally, imaging for high-risk disease may also be increasingly adopted given the push towards treating patients with neoadjuvant immunotherapy.

Despite the information available on imaging among patients with later stage melanoma, less is known about the use of pre-operative imaging in this high-risk cohort of patients with T3b-T4b melanoma and its impact on medical decision making. Therefore, we performed a retrospective analysis of the role of peri-operative cross-sectional imaging and its impact on medical decision making in the management of patients with T3b, T4a and T4b melanoma. We also sought to determine how imaging findings compare to additional staging information obtained from subsequent SLN biopsy.

Specifically, we hypothesized that our retrospective review would show an impact on medical decision making in patients who underwent pre-operative imaging, particularly in older patients, with an increase in the detection of regional and distant disease. Additionally, we expected to find that SLN biopsy would detect more regional nodal disease than cross sectional imaging, highlighting its continued importance in staging, even in T3b-T4b patients.

## Methods

### Data source and collection

The Yale-New Haven Hospital Melanoma Registry was queried for patients older than 18 years of age who presented with T3b, T4a or T4b melanoma and who had undergone a wide local resection between January 1, 2005 and December 31, 2020. This is inclusive of patients found on pre-operative imaging to have metastatic disease, as at our institution the primary melanoma is removed in nearly all cases even in stage IV disease. In addition, all patients with at least stage IIB received imaging at our institution during the study period. The clinical reason for timing and imaging selection was not available. This study was approved by the Yale University Institutional Review Board.

In combination with the data queries from a prospectively maintained Melanoma Registry, a retrospective chart review was performed for each patient. Electronic medical records were reviewed to validate data gathered from the registry and to collect additional information including: 1) patient covariates: age (grouped based on data distribution) and sex; 2) disease characteristics: location (defined as head/neck, trunk, extremities), final pathologic stage (based on AJCC 8^th^ edition), histological type (defined as acral lentiginous, desmoplastic, nodular, superficial spreading, unknown), Breslow thickness (defined as < 4mm, 4–8mm, > 8mm; grouped based on data distribution and to reflect the advanced staging of the population), mitotic rate (0–2 mm^2^, 3–5 mm^2^, >=6 mm^2^) and presence of ulceration (defined as presence or absence); 3) treatment and management modalities: receipt and findings of SLN biopsy (performed at the same time as the wide local resection) and completion lymph node dissection (CLND; performed before or after post-operative imaging), timing of cross-sectional imaging (defined as within 4 months of surgery pre-operative, or post-operative defined as within 4 months after surgical resection), findings of cross-sectional imaging (defined as in-transit disease, nodal disease, metastatic disease, incidental cancer, other (further defined as incidental findings such as kidney stones, nodules and soft tissue masses)) and receipt of immune or targeted therapy; and 4) long-term data: recurrence, cause of death, date of death and date of last follow-up.

### Study population

**Figure 1:**
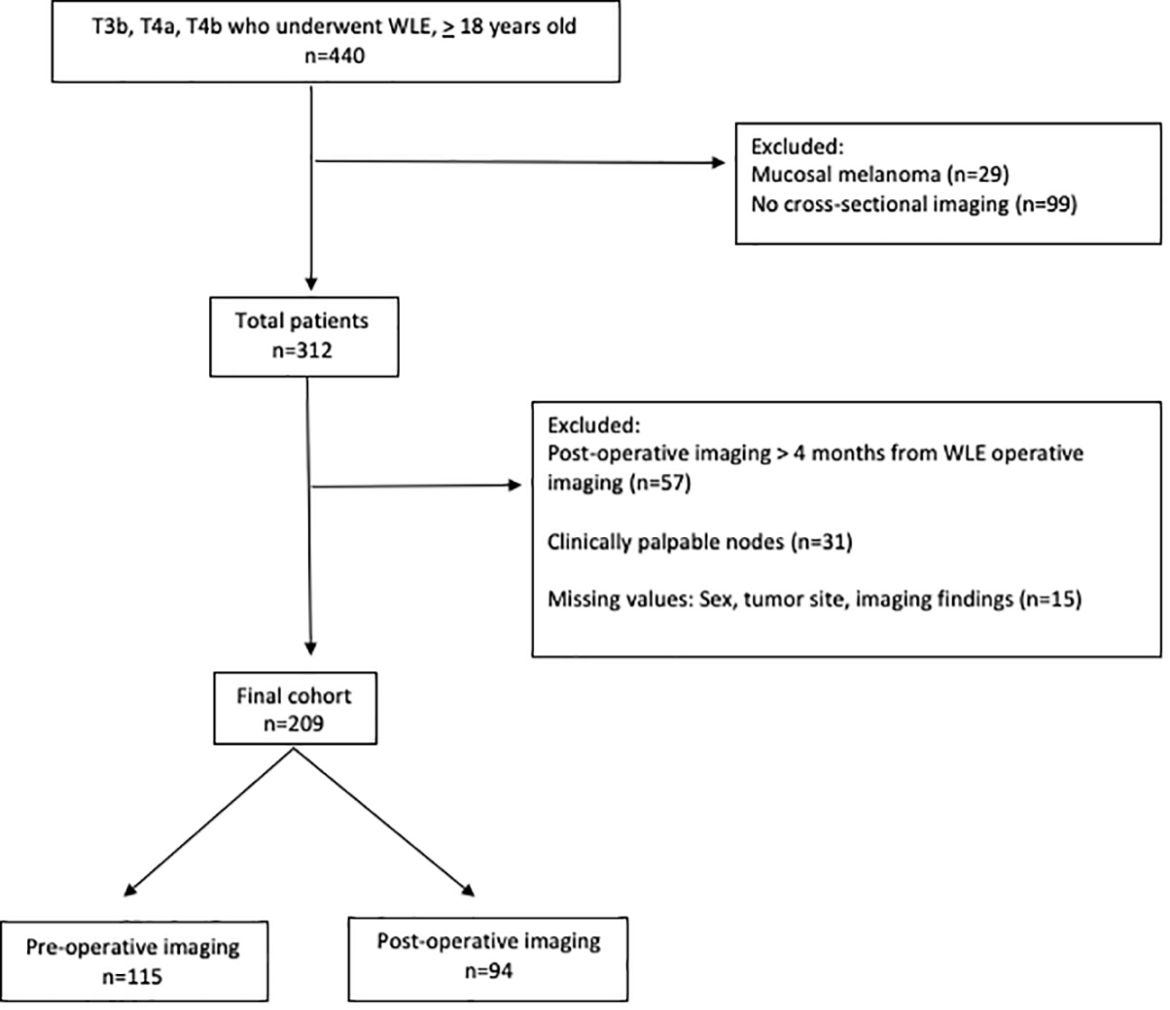
Patient selection criteria.

Patients diagnosed with melanoma with a clinically negative nodal exam who underwent wide local excision between 1/1/2005 and 12/31/2020 at age 18 or older were included. Our institutional practice is to typically excise the primary even in the setting of additional disease allowing for identification of the appropriate patient cohort for study. Patients who underwent cross-sectional imaging (defined as PET, CT, MRI) pre-operatively or post-operatively were included in the final cohort. Patients with mucosal melanoma were excluded (n=29) as this subtype is clinically managed differently at our institution, as compared to the other subtypes. All patients with clinically palpable nodes were excluded (n=31). The following missing or unknown values were excluded: sex, tumor site and imaging findings (n=15). The patient selection schema can be found in **Figure 1**. As such, we only included patients with complete data.

### Exposure

The patient cohort was divided into two groups for comparison: patients who underwent pre-operative imaging and patients who underwent post-operative imaging.

### Outcomes

The primary outcome was positive findings on pre-operative or post-operative imaging, defined as in-transit or nodal disease, metastatic melanoma, or incidental cancer. These were considered positive based on radiographic findings alone. Secondary outcomes included recurrence and recurrence free survival (RFS) (defined from the date of surgery).

### Statistical analysis

Summary statistics were reported as percentages for categorical variables and as medians with interquartile range (IQR) for continuous variables. Categorical variables were compared using the Chi Square or Fisher’s Exact Test and continuous variables were compared using Wilcoxon rank-sum test. Propensity scores with nearest neighbor matching were created for the odds of undergoing pre-operative imaging, adjusted for age, sex, ulceration, Breslow depth, mitotic rate and receipt of systemic therapy. Using the matched cohorts, overall recurrence free survival from the date of surgery was analyzed using the Kaplan-Meier method. A sub-analysis was performed in a group of patients 60 years of age or older to evaluate primary and secondary outcomes. Statistical significance was set at >= 0.05. All data analyses were conducted with SAS 9.4 (SAS Institute, Cary, NC).

## Results

### Patient characteristics

A total of 209 patients were identified (**Table 1**). In the overall cohort, the median age at diagnosis was 65 (IQR 54, 76) and 65.1% were male. Patients studied were more likely to have T4b disease at the time of presentation on initial biopsy (47.9%), to present with nodular melanoma (39.7%) and to have a primary melanoma on the extremity (44.0%).

**Table 1:** Patient demographics.

	Total Cohort n=209	Pre-Operative n=115	Post-Operative n=94	p-value
Age				0.3863
<50	38 (18.2%)	22 (19.1%)	16 (17.0%)	
50-60	41 (19.6%)	20 (17.4%)	21 (22.3%)	
60-70	46 (22.0%)	23 (20.0%)	23 (24.5%)	
70-80	46 (22.0%)	24 (20.9%)	22 (23.4%)	
>80	38 (18.2%)	26 (22.6%)	12 (12.8%)	
Sex				0.2637
Male	136 (65.1%)	71 (61.7%)	65 (69.2%)	
Female	73 (34.9%)	44 (38.3%)	29 (30.9%)	
T stage				0.1026
T3b	50 (23.9%)	22 (19.1%)	28 (29.8%)	
T4a	59 (28.2%)	38 (33.0%)	21 (22.3%)	
T4b	100 (47.9%)	55 (47.8%)	45 (47.9%)	
N Stage				0.0569
N0	113 (54.1%)	69 (60.0%)	44 (46.8%)	
N1-3	96 (45.9%)	46 (40.0%)	50 (53.2%)	
Histology				0.0857
Acral Lentiginous	15 (7.2%)	13 (11.3%)	2 (2.1%)	
Desmoplastic	14 (6.7%)	9 (7.8%)	5 (5.3%)	
Nodular	83 (39.7%)	45 (39.1%)	38 (40.4%)	
SSM	55 (26.3%)	28 (24.4%)	27 (28.7%)	
Unknown	42 (20.1%)	20 (17.4%)	22 (23.4%)	
Tumor Site				0.8005
Extremities	92 (44.0%)	53 (46.1%)	39 (41.5%)	
Head/Neck	47 (22.5%)	25 (21.7%)	22 (23.4%)	
Trunk	70 (33.5%)	37 (32.2%)	33 (35.1%)	
Ulceration	146 (69.9%)	75 (65.2%)	71 (75.5%)	0.1060
Breslow Depth (mm)				0.0260
<4	49 (23.4%)	19 (16.5%)	30 (31.9%)	
4-8	110 (52.6%)	68 (59.1%)	42 (44.7%)	
>8	50 (23.9%)	28 (24.4%)	22 (23.4%)	
Mitotic Rate (mm^2^)				0.7759
0-2	58 (27.8%)	29 (25.2%)	29 (30.9%)	
3-5	53 (25.4%)	30 (26.1%)	23 (24.5%)	
>6	86 (41.2%)	50 (43.5%)	36 (38.3%)	
Unknown	12 (5.7%)	6 (5.2%)	6 (6.4%)	
Receipt of systemic therapy	113 (54.1%)	57 (49.6%)	56 (59.6%)	0.1486
Imaging Type				0.1176
CT	110 (52.6%)	59 (51.3%)	58 (61.7%)	
PET/PET-CT	88 (42.1%)	55 (47.8%)	33 (35.1%)	
MRI	4 (1.9%)	1 (0.9%)	3 (3.2%)	
Sentinel Lymph Node Biopsy Completed	162 (77.5%)	92 (80.0%)	70 (74.5%)	0.3406
Positive Sentinel Lymph Node	77 (47.5%)	33 (42.9%)	44 (57.1%)	0.0007
Completion Lymph Node Dissection Completed	43 (20.6%)	16 (13.9%)	27 (28.7%)	0.0084

When comparing patients who underwent pre-operative (n=115, 55.0%) versus post-operative imaging (n=94, 45.0%), there were patient selection differences based on Breslow depth. Patients with tumor depth less than 4 mm underwent pre-operative imaging less frequently (16.5% versus 31.9%), post-operative while those with a depth of 4-8 mm underwent pre-operative imaging more frequently (59.1% versus 44.7%) post-operative (p=0.0260). More patients who underwent pre-operative imaging were over age 80 (22.6%) as compared to those who underwent post-operative imaging (12.8).

### Imaging findings

**Figure 2:**
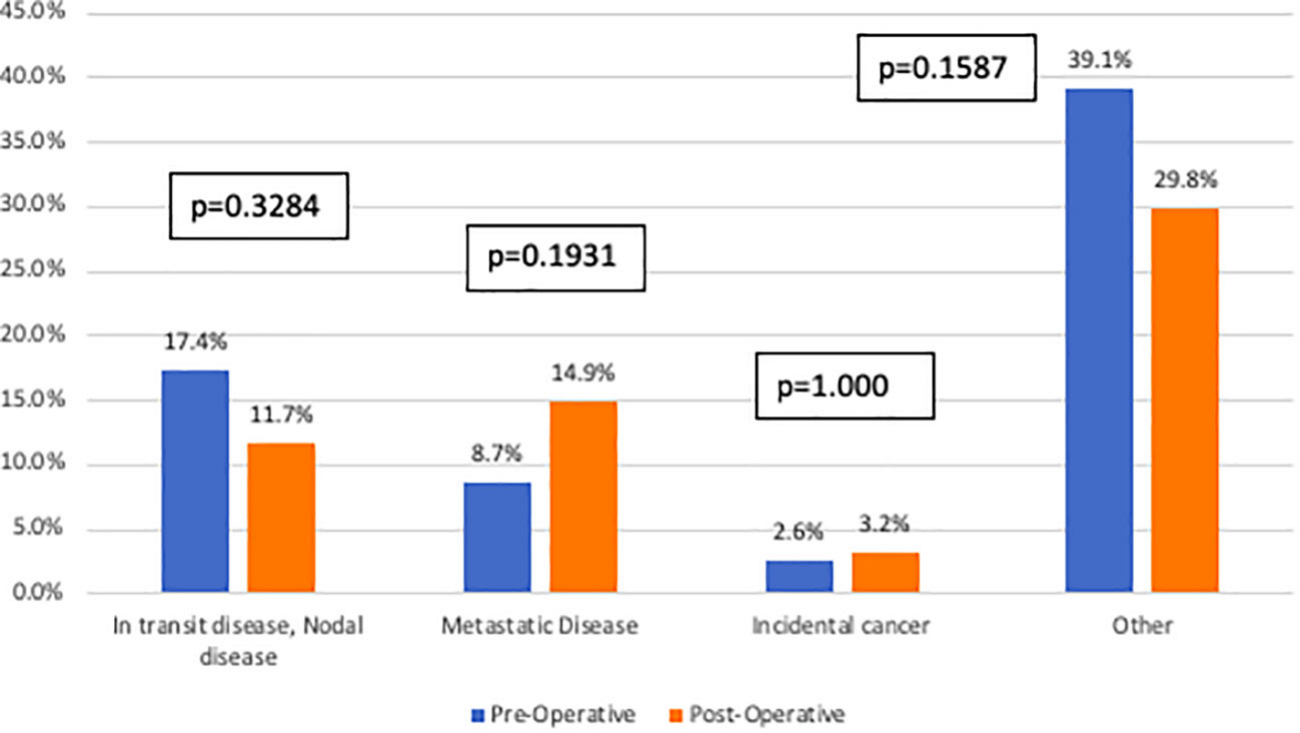
Imaging findings stratified by imaging cohort. This figure compares specific findings (defined as in-transit or nodal disease, metastatic disease, incidental cancer or other) between the pre-operative imaging cohort (blue) and the post-operative imaging cohort (orange). There were no statistically significant differences identified between the two groups.

In total, 109 patients (52.2%) had positive findings on imaging: 57.8% pre-operatively and 42.2% post-operatively (p=0.3999). In the total cohort, no statistically significant differences were found between the two imaging cohorts based on the identification of in-transit or nodal disease (pre-operative 17.4% versus post-operative 11.7%, p=0.3284), metastatic disease (pre-operative 8.7% versus post-operative 14.9%, p=0.1931), incidental cancer (pre-operative 2.6% versus post-operative 3.2%, p=1.000), or other findings (pre-operative 39.1% versus post-operative 29.8%, p=0.1587) (**Figure 2**). Sub-analyses were performed based on sex, tumor site, histology, Breslow depth, mitotic rate, and ulceration. No differences were identified in pre-operative and post-operative imaging findings, but this may be in part due to small sample size.

### Sentinel node biopsy

Overall, 77.5% of patients (n=162) underwent sentinel node biopsy with 42.9% (33/92) positive in the pre-operative group and 57.1% (44/70) positive in the post-operative group (p=0.0007). In addition, patients who underwent a completion lymph node dissection underwent post-operative imaging at a higher proportion (28.7% versus pre-operative 13.9%, p=0.0084). Similarly, of patients who underwent both SLNB and CLND, a greater proportion had post-operative imaging (**Supplemental Table 1**).

### Recurrence and survival

**Figure 3:**
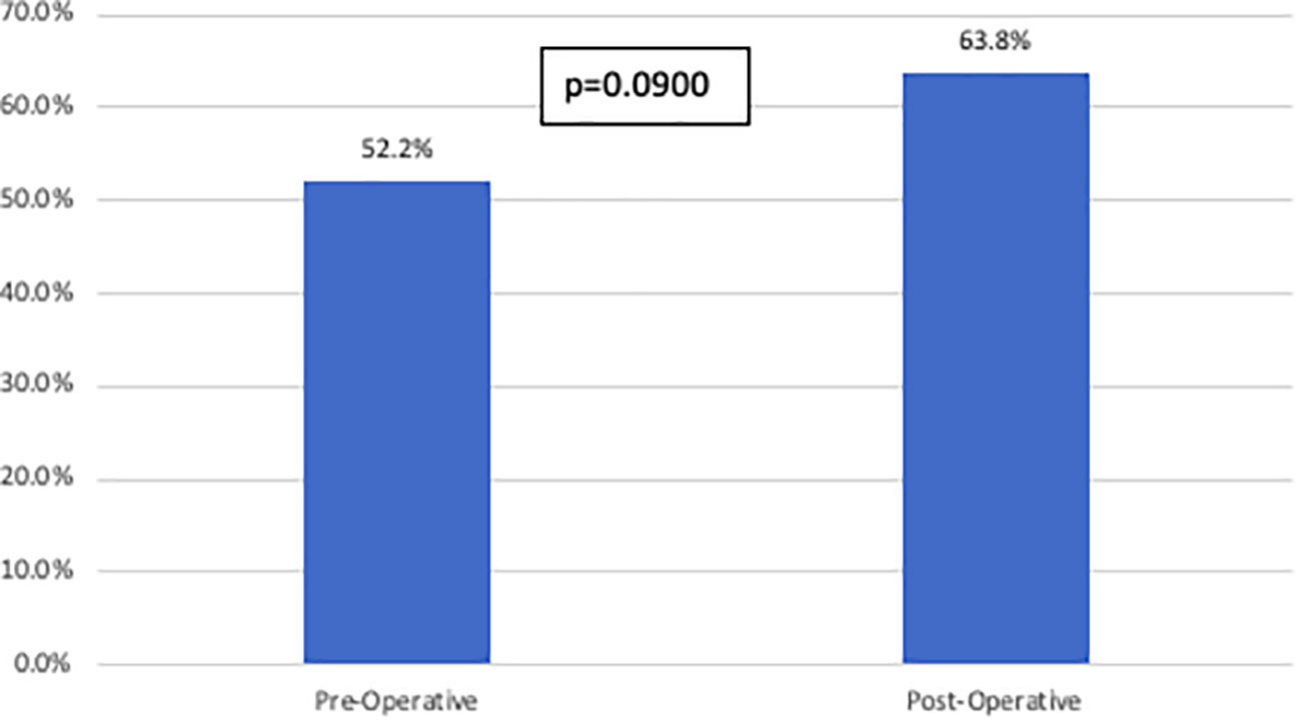
Recurrence rates stratified by imaging cohort. This figure compares overall recurrence rates between the pre-operative imaging cohort and the post- operative imaging cohort. There was no statistically significant difference identified between the two groups.

In the total cohort, 57.4% of patients ultimately recurred. There was no difference in recurrence patterns based on imaging cohort (**Figure 3**).

**Figure 4:**
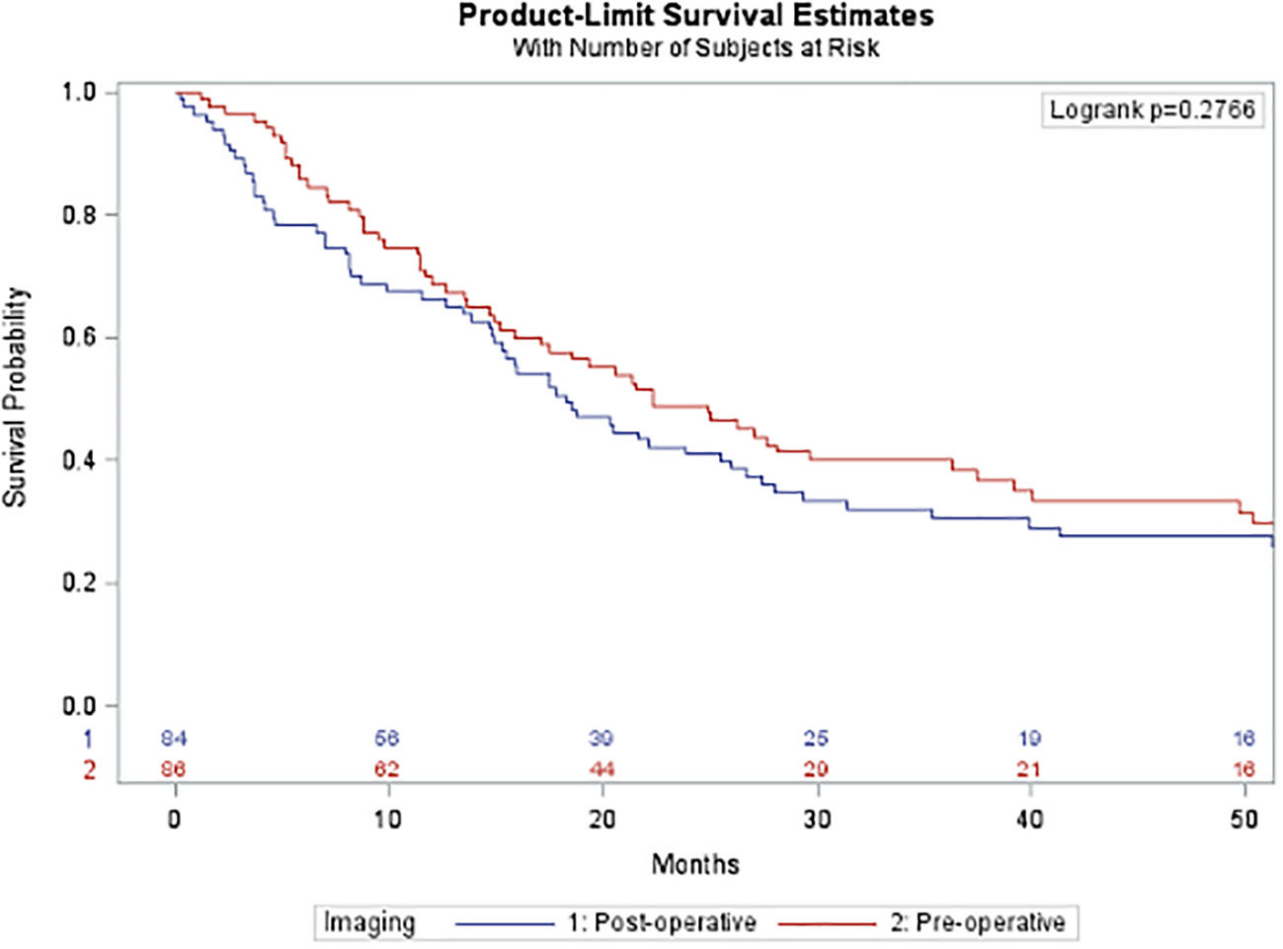
Overall recurrence free survival after propensity-score matching. This figure compares overall recurrence free survival after propensity score matching between the pre- operative imaging cohort (blue) and the post-operative imaging cohort (red). There was no statistically significant difference in survival between the two groups.

After propensity score matching, there was no difference in overall recurrence free survival (pre-operative 22.3 months versus post-operative 18.2 months, p=0.2755; **Figure 4**) based on the timing of imaging.

### Sub-analysis based on age

In a sub-group analysis of patients greater than or equal to 60 years of age (n=130), similar imaging findings were identified: there was no difference in the proportion of patients found to have in-transit or nodal disease, metastatic disease, incidental cancer, or other findings, based on imaging cohort. Similarly, among patients with a positive SLNB, there was no difference between age cohorts of patients with positive pre-operative imaging.

After propensity score matching, there was no difference in recurrence free survival based upon timing of imaging (pre-operative 27.6 months versus post-operative 17.8 months, p=0.0656).

## Discussion

Our study demonstrates that pre-operative imaging is not associated with statistically significant differences that influence the peri-operative management of high-risk melanoma, including no recurrence-free survival benefit, or differences based upon age, in contrast to our original study hypotheses.

Previous studies have established the value of cross-sectional imaging, particularly PET and PET/CT, in the staging of malignant melanoma. It has been previously demonstrated that the use of PET/CT is highly diagnostic for the evaluation of N- and M-staging, response to therapy and recurrence (15–18). Additionally, a meta-analysis by Schroer-Gunther et al. demonstrated increasing prognostic accuracy with increasing stage in the use of cross-sectional imaging (19). Interestingly, this meta-analysis included randomized control trials composed of patients with all stages of melanoma, with cohort sizes ranging from 17 to 251. Despite reporting on imaging, the study was primarily focused on patient-relevant quality of life outcomes. Danielsen et al. evaluated 167 patients with a primary melanoma greater than 2mm in thickness or invasive melanoma no greater than 2mm in thickness with at least one high-risk histological feature and/or had already undergone wide local excision. The authors found among those with staging PET/CT positivity for metastatic melanoma (19.2%), predictors included lymphadenopathy, bleeding from the primary tumor, SLN status, mitotic rate, tumor thickness, Clark level, male gender eye color and history of blistering sunburns (13). However, they did not dichotomize when imaging was obtained as we studied.

Few studies have explored the impact of imaging in the pre-operative period in patients who have high risk primary melanoma, with clinically node negative disease. Frary et al. evaluated 47 patients in Denmark with a positive SLNB who underwent FDG-PET/CT pre-operatively prior to lymph node dissection to attempt to find distant metastases. Ultimately, this diagnostic strategy did not result in significant findings, but rather a false positive finding in 13% of patients (20). Similarly, Wagner et al. found that among 144 early-stage melanoma patients, pre-operative FDG-PET imaging did not impact patient care (12).

Our study adds to the current literature by evaluating the role of pre-operative imaging in high-risk melanoma in one of the largest reported cohorts studied. Ultimately, we found no difference in the identification on imaging for in-transit or nodal disease, metastatic disease, incidental cancer, or other findings when imaging was obtained preoperatively when compared to post-operative imaging. We had hypothesized, particularly among our high-risk patient cohort with a high proportion of T4b at the time of diagnosis, that imaging would impact management and ultimately recurrence free survival. Despite not finding a statistically significant difference between the imaging of timing on imagine, this does not discount the value that imaging plays overall in this population. We may have not been able to find a statistically significant difference due to the high incidence of impactful imaging findings in both patient cohorts, with in-transit or nodal disease found in 17.4% of patients pre-operatively and 11.7% post-operatively. This is in addition to the identification of metastatic disease found pre-operatively in 8.7% of patients and 14.9% post-operatively. This amounts to imaging findings influencing care decisions in one out of every four patients, in either cohort, and warrants continued investigation.

Our findings cannot be interpreted without the acknowledgement of the continuing role of SLNB and to a lesser extent CLND. Overall, 77.5% of patients underwent SLNB and 20.6% went on to a CLND in the time prior to the MSLT-II study. Importantly, in the setting of clinically node negative disease, with negative pre-operative imaging, nearly 43% of patients in this high-risk group were found to have involvement on sentinel node biopsy. This is critical to note as it demonstrates that imaging alone cannot replace the role of SLNB, which provides invaluable information in the management and subsequent counseling of the risks and benefits of adjuvant therapy based upon accurate staging. This comes at a critical time as these patients are now eligible for adjuvant treatment with immunotherapy even without undergoing sentinel node biopsy (14). This makes understanding the impact of pre-operative versus post-operative imaging that much more salient.

Lastly, we chose to perform a sub-group analysis in an older cohort of the study population. There is increasing evidence that there are changes in the rate of sentinel node positivity in older versus young patients despite increased risk of melanoma specific death (6, 7, 21). This may be attributable to age related changes in dermal lymphatics, ultimately rendering older patients at higher risk for metastatic disease than regional node disease (8, 22, 23). With that in mind, it could be hypothesized that this group would be more likely to have positive findings in the pre-operative period. Ultimately, we did not find any differences in imaging or recurrence free survival in this cohort. However, one could still posit that for the elderly or highly morbid population, that the confirmation of an absence of additional disease would potentially be of value before final decision making is pursued.

Our study has several important limitations. The retrospective nature of our study, in combination with a small sample size, may introduce bias. In addition, our cohort only includes patients who underwent wide local incision. Therefore, this may present a selection bias as we do not have information on those who did not undergo surgery at all. However, the practice at our institution is to typically excise the primary even in the setting of metastatic disease, so we do feel that we have captured most of these at-risk patients. In addition, the information gained following surgery, such as a more accurate tumor thickness, may introduce a selection bias. Similarly, our cohort does not include patients who received neoadjuvant therapy, and therefore our findings are not generalizable to that population. Similarly, we ultimately did not adjust for additional therapies, which may have impacted recurrence free survival, particularly among patients who were upstaged. In addition, our outcomes are based on radiographic data only and are not confirmed by biopsy results. This is further limited as our radiographic data does not include ultrasound, which can be a useful modality when assessing lymph node status. We attempted to mitigate this by only including patients with clinically negative node examinations. Importantly, we do not have granular information on the decision-making process for when an image was obtained, which could provide a more comprehensive understanding of our results. Lastly, our study was performed at a single institution which ultimately limits the generalizability of the results.

## Conclusions

Pre-operative cross-sectional imaging when compared to imaging obtained post-operatively does not identify a statistically significantly increased change in the management of patients with T3b-T4b melanoma. This appears true in both the perioperative period, regarding surgical management, and long term, regarding recurrence free survival. More so, this holds true across all patient ages. Considering recent changes to adjuvant therapy guidelines and the increasing use of cross-sectional imaging, careful consideration of timing and patient benefit is critical. It also highlights the importance of including sentinel node biopsy to appropriately stratify patients for staging to allow for appropriate surveillance and decisions regarding adjuvant therapy.

## Data availability statement

The raw data supporting the conclusions of this article will be made available by the authors, without undue reservation.

## Author contributions

Conception & Design - MP, RM, SW, JC, KO Data collection & Organization - MP, RM, AK, VL, RB, KO Data Analysis & Interpretation - MP, KO Manuscript drafts - MP, RM, KO Manuscript revision & editing - All authors. All authors contributed to the article and approved the submitted version.
