# Supplementary material for: The role of imaging and sentinel lymph node biopsy in patients with T3b-T4b melanoma with clinically negative disease
Source: Front Oncol. 2023 May 8;13:1143354. doi: 10.3389/fonc.2023.1143354 (PMC10200883; doi:10.3389/fonc.2023.1143354)
Supplement: Supplementary file 1 [file Table_1.pdf]

Supplemental Table 1. The distribution of sentinel lymph node biopsy and completion lymph node dissection, stratified by pre and post-operative imaging

	**Pre-operative imaging**	**Post-operative imaging**
**SLNB alone**	76	43
**SLNB & CLND**	16	27
**CLND alone**	1	1
